# Correlation between serum GDF-15 level and pulmonary vascular morphological changes and prognosis in patients with pulmonary arterial hypertension

**DOI:** 10.3389/fcvm.2023.1085122

**Published:** 2023-05-23

**Authors:** Yasenjiang Maimaiti, Hui Cheng, Zitong Guo, Xiaolin Yu, Adilijiang Tuohuti, Guoqing Li

**Affiliations:** ^1^ Gerontology Center, People's Hospital of Xinjiang Uygur Autonomous Region, Urumqi, China; ^2^ Department of Cardiology, People's Hospital of Xinjiang Uygur Autonomous Region, Urumqi, China

**Keywords:** intravascular ultrasound, growth differentiation factor-15 (GDF-15), mortality rate, pulmonary hypertension, pulmonary vascular morphology

## Abstract

**Objective:**

To investigate how serum GDF-15 concentration affects pulmonary artery hemodynamics and pulmonary vascular morphological changes in patients with pulmonary arterial hypertension.

**Methods:**

A total of 45 patients admitted to our hospital from December 2017 to December 2019, were selected for the study. Pulmonary vascular hemodynamics and pulmonary vascular morphology were detected by RHC and IVUS. Serum GDF-15 levels were detected by enzyme-linked immunosorbent assay (ELISA). Based on the concentration of GDF-15, the patients were divided into two groups—the normal GDF-15 group (GDF-15 <1,200 pg/ml, 12 cases) and the elevated GDF-15 group (GDF-15 ≥1,200 pg/ml, 33 cases). A statistical analysis was performed to compare the effects of normal blood GDF-15 levels and high serum GDF-15 levels on hemodynamics and pulmonary vascular morphology in each group of patients.

**Results:**

The average levels of RVP, sPAP, dPAP, mPAP, and PVR in patients with elevated GDF-15 levels were higher than those in patients with normal GDF-15 levels. The difference between the two groups was statistically significant (*P* < 0.05). The average levels of Vd, elastic modulus, stiffness index β, lesion length, and PAV in the normal GDF-15 group were lower than those in the elevated GDF-15 group. The average levels of compliance, distensibility, and minimum l umen area were higher than those in the elevated GDF-15 group. The difference between the two groups was statistically significant (*P* < 0.05). The survival analysis results showed that the 1-year survival rate of patients with normal GDF-15 levels and elevated GDF-15 levels was 100% and 87.9%, respectively, and that the 3-year survival rate of patients with normal GDF-15 levels and elevated GDF-15 levels was 91.7% and 78.8%, respectively. The survival rates of the two groups were compared by the Kaplan Meier method, and the difference was not statistically significant (*P* > 0.05).

**Conclusion:**

Patients with pulmonary arterial hypertension with elevated GDF-15 levels have higher pulmonary arterial pressure, higher pulmonary vascular resistance, and more serious pulmonary vascular lesions, which are potentially more harmful. There was no statistically significant difference in survival rates among patients with different serum GDF-15 levels.

## 1. Background

Pulmonary hypertension (PH) is a group of fatal diseases characterized by abnormally elevated pulmonary arterial pressure (PAP) and pulmonary vascular resistance (PVR), ultimately leading to right heart failure and death. Among them, pulmonary arterial hypertension (PAH) is the most fatal type of PH (1, 2). At present, the median survival time of PAH patients treated with PAH drugs is only 7 years (3). The disease progresses rapidly, seriously affects the patient's life quality, and has a poor prognosis. PAH is a common complication in patients with connective tissue diseases. PAH associated with connective tissue diseases (PAH-CTD) mainly includes systemic lupus erythematosus (SLE)-associated PAH, systemic sclerosis (SSc), mixed connective tissue diseases (MCTD), rheumatoid arthritis (RA), and Sjögren's syndrome (4). The diagnosis and evaluation methods of PAH mainly include right heart catheterization (RHC), lung biopsy, and pulmonary angiography. Right heart catheterization (RHC) is still the “gold standard” for evaluating and diagnosing PAH. However, it can't detect abnormal pulmonary vascular performance, nor can it distinguish slight changes in pulmonary vascular performance among different types of PAH (5). A lung biopsy can accurately evaluate pulmonary vascular lesions, but its clinical application is limited because of the heterogeneity of pulmonary vascular lesions in PAH patients and the relatively invasive nature of biopsy. Intravascular ultrasound (IVUS) is a new imaging technology that combines non-invasive ultrasound with invasive cardiac catheterization. It is widely used in the study of coronary heart disease (6). However, a large number of studies done in China and overseas, have shown that IVUS can not only evaluate the morphological changes of pulmonary blood vessels but it can also quantitatively and qualitatively analyze the morphological and functional changes of pulmonary blood vessels. It plays an important role in evaluating the morphological changes and detecting mechanical properties of pulmonary blood vessels in PAH (7–10). However, it is an invasive procedure, which is difficult to perform, and there are few studies on the correlation between pulmonary artery IVUS performance and the prognosis of PAH patients.

Growth differentiation factor-15 (GDF-15), a member of the transforming growth factor-β cytokine superfamily, was a stress response protein, which plays a role in anti-apoptosis, anti-inflammation, and protection of vascular endothelium in the body (11, 12). GDF-15 is mainly involved in various biological processes such as tissue repair, and organ growth regulation and differentiation, and has also been implicated in the progression of many diseases (13, 14). It has been reported that GDF-15 regulates the process of vascular proliferation, differentiation, remodeling, and inflammatory damage repair mainly through anti-inflammation, anti-apoptosis, anti-fibrosis, and anti-oxidation. In mononuclear macrophages, angiotensin II, interleukin-1, and tumor necrosis factor can all induce the production of GDF-15, and exert its anti-inflammatory effect by activating macrophages. As a protective factor, the GDF-15 protein is secreted extracellular and binds to the TGF-β family of receptors on the cell surface, enhancing the activation of intracellular Smad4 signaling pathway by promoting intracellular Smad2/3 and Smad1/5/8 phosphorylation signals, and ultimately inhibiting cell growth (11). On the other hand, GDF-15 can also function through a non-Smad-dependent pathway. Under some pathological conditions, GDF-15 inhibits the pro-proliferation effect of Akl/EliK by blocking the activation of epidermal growth factor receptor, and can also inhibit the oxygen free radical mediated NF-kB/JNK signal activation by activating PI3K/Akt/eNOS signaling pathway, thus protecting vascular endothelial cells from serious damage (12). Studies have shown that, as an important cardiovascular protective factor, GDF-15 is closely related to the diagnosis and prognosis of many cardiovascular diseases (15). Studies have found that the expression of GDF-15 in the pulmonary tissue of PAH patients is significantly higher than that in patients with normal pulmonary tissue and it is mainly concentrated in pulmonary vascular endothelial cells (16). Meadows et al. showed that the level of GDF-15 in the plasma of PAH patients with systemic sclerosis (SSc) is significantly higher than that of SSc patients without pulmonary arterial hypertension (17). Kempf et al. found that GDF-15 can be induced by the expression of cardiomyocytes in the condition of ischemia reperfusion through a nitric oxide-dependent pathway, and GDF-15 can also be induced by oxidative stress caused by other cardiovascular events (such as pressure overload, heart failure, atherosclerosis, etc.), thus inhibiting the hypertrophy and structural remodeling of cardiomyocytes (18). Therefore, GDF-15 may be a useful biomarker in PAH-CTD patients.

However, there are few reports on the relationship between GDF-15 level and morphological changes in pulmonary blood vessels of patients with PAH. GDF-15, a member of the TGF-β family, activates the TGF-β receptor. From this point of view, GDF-15 has a dual role, that is, it is involved in the initiation of the body's self-protective response, and in some cases plays a protective role. For example, initiation of autophagy, inhibition of inflammation, inhibition of apoptosis, etc. (19–25). However, in some cases, it plays a bad role, such as tissue fibrosis and other diseases (26, 27). According to existing studies, the expression level of GDF-15 in pulmonary hypertension is indeed increased, and it is correlated with the survival rate of patients. Patients with low GDF-15 have a higher survival rate. But whether it protects or promotes damage is less clear.

In this study, we used an enzyme-linked immunosorbent assay (ELISA) to detect the level of GDF-15 in the serum of PAH patients. We also analyzed how GDF-15 is related to pulmonary artery hemodynamics and pulmonary vascular morphological changes, to explore whether GDF-15 could indirectly reflect the severity of pulmonary vascular morphological changes in PAH and further explore its influence on the mortality or prognosis of PAH patients.

## 2. Materials and methods

### 2.1. Study participants

Study participants were selected according to the established inclusion and exclusion criteria: Inclusion criteria: (1) The participants (or legal guardian) understand the test requirements and treatment procedures and sign a written informed consent prior to performing any examinations or procedures required by the protocol. (2) Male or female participants ≥18 years old and <80 years old. (3) Highly suspicious PAH after echocardiographic screening (SPAP ≥ 40 mmHg). Exclusion criteria: (1) Pregnant or lactating women. (2) The researchers believe that participants in the study are at increased risk [(a) patients with severe mental illness or congenital mental retardation, such as schizophrenia and depression; (b) patients with severe organ dysfunction such as heart, lung, liver, and kidney, severe endocrine impairment, severe infectious diseases, and toxic encephalopathy; (c) patients with stroke, Parkinson's disease, ischemic cerebrovascular disease, brain tumor, and other neurological diseases that can cause brain dysfunction; (d) patients with a history of severe head trauma and those who have been determined to be alcohol dependent or drug dependent within the past 6 months]. (3) Patients with mental disorders who are unable to sign informed consent, or who are unable to follow trial protocol. (4) Patients with severe infection, severe liver failure, renal insufficiency and severe bleeding tendency. (5) Patients with malignant tumors. (6) Complicated with second or more degrees of atrioventricular block, or pathological sinoatrial syndrome, hemodynamic instability, spastic bronchial asthma, and adenosine or adenosine triphosphate allergy. (7) Patients who cannot lie flat on the operating table. The study duration was from December 2017 to December 2019, a total of 2 years. We selected 60 patients from the People's Hospital of Xinjiang Uygur Autonomous Region who were highly suspected of PH, based on medical history and echocardiography screening. After inclusion, RHC and IVUS examinations were performed. According to RHC, laboratory examinations, and diagnosis by the Department of Cardiology, and Department of Rheumatology and Immunology of People's Hospital of Xinjiang Uygur Autonomous Region, 27 patients were diagnosed as PAH-CTD (PAH-CTD group), 18 patients were diagnosed as other types of PAH (10 cases of idiopathic pulmonary arterial hypertension, 4 cases of portal pulmonary arterial hypertension, 4 cases of congenital heart disease-related pulmonary arterial hypertension) (other types of PAH group), and 15 patients were diagnosed as without PAH (control group). According to the results of GDF-15 detection, the patients were divided into two groups: normal GDF-15 group (GDF-15 <1,200 pg/ml) and elevated GDF-15 group (GDF-15 ≥1,200 pg/ml). This study was approved by the Ethics Committee of People's Hospital of Xinjiang Uygur Autonomous Region (approval number:). All selected patients signed the informed consent.

### 2.2. RHC and IVUS examination

According to the European Heart Journal's guidelines for the diagnosis and treatment of pulmonary hypertension (28), RHC (8.5F, Baxter Healthcare, Edwards Critical Care Division, Deerfield, IL, USA) was performed to detect the pulmonary artery hemodynamics. Detection indicators include—right atrial pressure (RAP), right ventricular pressure (RVP), pulmonary artery systolic pressure (sPAP), pulmonary artery diastolic pressure (dPAP), mean pulmonary artery pressure (mPAP), pulmonary artery wedge pressure (PAWP), cardiac output/cardiac index (CO/CI), etc. The formula for PVR = (mPAP − PAWP)/CO. The formula for pulmonary arterial pulse pressure (PPP) = sPAP − dPAP. At the same time, we measured the blood oxygen saturation of the vena cava, right atrium, right ventricle, pulmonary artery, and systemic circulation.

IVUS examination was performed as soon as pulmonary artery pressure recovered after RHC and pulmonary artery angiography. A 40 MHz US catheter (Boston Scientific, US) with axial resolution of 43 µm was used to detect the lower pulmonary artery segment. As per Bressollette et al., the typical abnormality in pulmonary vascular performance is usually in the lower lung, and the degree of abnormality is similar between the left and right lungs (29). Therefore, four pulmonary segments were detected (two segments from the left lower pulmonary arteries and two segments from the right lower pulmonary arteries) and the mean value was used for statistical analysis.

After the US catheter was sent to the distal end of the pulmonary artery, it was retracted to the proximal end of the pulmonary artery at a speed of 0.5 mm/s. The image data were read using iLabTM system (Boston Scientific, USA) to ensure that all the images were of good quality (a complete circumferential boundary existed between the tunica media and the medial wall of tunica adventitia of pulmonary blood vessels). The image data were recorded and saved on a Sony DVD.

### 2.3. Pulmonary vascular performance measurement and calculation

Using a single-blind method, two experienced operators independently measured all clinical and hemodynamic data using imap software (ImageJ Ver 1.44, NIH, USA). All pulmonary blood vessels were divided into 2 segments: the distal segment with diameter <5 mm and the proximal segment with diameter >5 mm. A total of 480 pulmonary blood vessel segments were measured (216 from PAH-CTD group, 140 from other types of PAH group, and 120 from control group).

Since IVUS cannot distinguish the tunica adventitia of pulmonary blood vessels, in this study, we took the data of the inner diameter limits of pulmonary blood vessels (including tunica intima and tunica media of pulmonary blood vessels). The data included total area of blood vessels (tunica intima and tunica media of pulmonary blood vessels) in diastole and systole (VAd and VAs), vascular diameter (VDd and VDs), lumen area (LAd and LAs), lumen diameter (LDd and LDs) and minimum lumen diameter (MLDd and MLDs), average vascular diameter = (VDd + VDs)/2, mean wall thickness (MWT, Mean wall thickness) = [(VDd + VDs)/2 − (LDd + LDs)/2]/2, and the mean wall thickness percentage (WTP, percentage of MWT) = (2 × MWT) × 100%/VD. If the WTP of the distal segment of the pulmonary artery of PAH patients was higher than that of the proximal segment, they were included in the distal remodeling group (*n* = 21), and vice versa; if the WTP of the proximal segment of pulmonary artery of PAH patients was higher than that of the distal segment, they were included in the proximal remodeling group (*n* = 24).

Pulmonary vascular mechanical performance indicators include compliance, distensibility, elastic modulus, and stiffness index β (28–32). The calculation methods of each index are as follows: compliance = (VAd − VAs) × 100/PPP; distensibility degree = (VAd − VAs) × 100%/PPP × VAd; elastic modulus = PPP × VDd/(VDd − VDs); stiffness index β = Ln(sPAP/dPAP)/[(VDd − VDs)/VDd].

### 2.4. Laboratory examination

Blood samples were collected for GDF-15 and NT-proBNP measurement during the first right heart catheterization. Blood was collected from the healthy control group in the morning. The whole blood was centrifuged at 3,500 g for 15 min and the supernatant was collected as serum, then frozen and stored at −80°C for further analysis.

### 2.5. Enzyme-linked immunosorbent assay (ELISA)

GDF-15 and NT-proBNP were detected using the Elisa kit (CUSABIO) according to the instructions. Standard laboratory techniques measured creatinine and uric acid. Serum samples are measured directly according to the kit instructions. The concentration of GDF-15 in human samples was calculated by standard curve and OD value.

### 2.6. Drug therapy and follow-ups

All patients were diagnosed by right heart catheterization, IVUS, and FFR examination, combined with various laboratory tests. Then, PAH patients received targeted drug therapy, including endothelin receptor antagonist (bosentan, phosphodiesterase inhibitor), sildenafil, tadalafil, etc. PAH-CTD patients were given connective tissue etiological treatment and targeted drug therapy concurrently. Patients were followed up every 1 month after discharge and every 3–6 months after half a year. Follow-ups included investigation of the recent condition of the patient, blood biochemical examination, concomitant medication, any hospitalization due to changes in patient condition. The primary mode of follow-ups was outpatient care. The last follow-up was by telephone. The clinical endpoint was all-cause mortality of patients.

### 2.7. Statistical methods

SPSS 25.0 was used for statistical analysis. R 4.2.1 was used to draw statistical charts. The measurement data are expressed by mean ± standard deviation $\left(\bar{x}\pm s\right)$, and *t*-test was used for comparison between two groups. Categorical variables were presented as percentages with Chi-squared test for comparison between two groups. Spearman correlation analysis was performed to analyze the correlation between serum GDF-15 level and each parameter. The 1- and 3-year cumulative survival rates were calculated by Kaplan-Meier analysis and compared with log-rank test. The main endpoint was all-cause mortality. Two-sided *P* < 0.05 is statistically significant.

## 3. Results

### 3.1. General clinical baseline data of patients

As shown in Table 1, there was no statically significant difference in baseline data such as age, sex, diabetes, hypertension, and coronary heart disease between GDF-15 <1,200 pg/ml group and GDF-15 ≥1,200 pg/ml group. The two groups of patients were comparable, as shown in Table 1.

**Table 1:** Baseline data of patients in each group.

Index	GDF-15 <1,200 pg/ml Group (*n* = 12)	GDF-15 ≥1,200 pg/ml Group (*n* = 33)	t/χ^2^	*P*
Age	47.42 ± 12.90	44.52 ± 15.70	0.572	0.570
**Gender**				
Male	6 (50.0)	21 (63.6)	0.682	0.409
Female	6 (50.0)	12 (36.4)		
Diabetes	3 (25.0)	4 (12.1)	1.111	0.292
Hypertension	2 (16.7)	11 (33.3)	0.517	0.472
Coronary heart disease	3 (25.0)	7 (21.2)	0.000	1.000
ALT (U/L)	28.5 ± 9.3	29.9 ± 8.7	−0.471	0.640
AST (U/L)	24.5 ± 6.2	21.3 ± 6.6	1.453	0.153
BUN (mmol/L)	7.3 ± 2.3	6.9 ± 2	0.581	0.564
CR (mmol/L)	75.1 ± 22.4	74.1 ± 19.5	0.147	0.884
UA (µmol/L)	357.5 ± 97.4	382.7 ± 95.1	−0.780	0.440
TC (nmol/L)	3.8 ± 0.9	4.1 ± 0.9	−0.934	0.356
TG (nmol/L)	1.5 ± 0.6	1.6 ± 0.7	−0.421	0.676
HDL (nmol/L)	1.2 ± 0.2	1.1 ± 0.3	1.595	0.118
LDL (nmol/L)	2.3 ± 0.6	2.6 ± 0.7	−1.085	0.284

ALT, alanine transaminase; AST, aspartate transaminase; CR, creatinine; UA, urea nitrogen; TC, total cholesterol; TG, triglyceride; HDL, high density lipoprotein cholesterol; LDL, low density lipoprotein cholesterol.

The PAH-CTD group had 14 patients with systemic lupus erythematosus (SLE), 4 patients with systemic sclerosis, 2 patients with Sjögren's syndrome, 1 patient with mixed connective tissue disease, and 1 patient with rheumatoid arthritis. The most common disease was SLE, accounting for 52% (14/27). The other types of PAH group had 10 patients with idiopathic pulmonary arterial hypertension (IPAH), 4 patients with portal pulmonary arterial hypertension, and 4 patients with congenital heart disease-related pulmonary arterial hypertension. Among them, IPAH patients were the largest number, accounting for 67% (12/18). In the control group, one patient had hepatopulmonary syndrome and the other patients had connective tissue disease.

### 3.2. Correlation analysis between GDF-15 and pulmonary artery hemodynamic parameters

The average levels of RAP and PAWP were not statistically significant in the GDF-15 <1,200 pg/ml and GDF-15 ≥1,200 pg/ml (*P* > 0.05) groups. The average levels of RVP, sPAP, dPAP, mPAP, CO, and PVR in GDF-15 ≥1,200 pg/ml group were higher than those in GDF-15 <1,200 pg/ml group. The difference between these two groups was statistically significant (*P* < 0.05), as shown in Table 2. Spearman's correlation analysis results showed that the level of GDF-15 was positively correlated with RVP, sPAP, dPAP, mPAP, CO, and PVR (*P* < 0.05), as shown in Table 3.

**Table 2:** Hemodynamic characteristics of patients in each GDF-15 group $\left(\bar{x}\pm s\right)$.

Index	GDF-15 <1,200 pg/ml Group (*n* = 12)	GDF-15 ≥1,200 pg/ml Group (*n* = 33)	*t*	*P*
RAP (mmHg)	6.9 ± 5.9	8.9 ± 3.6	1.378	0.176
RVP (mmHg)	25.3 ± 5.4	35.8 ± 9.8	3.506	0.001*
sPAP (mmHg)	53.6 ± 10.8	69.5 ± 16.4	3.110	0.003*
dPAP (mmHg)	26.8 ± 8.3	39.4 ± 9.9	3.928	0.000*
mPAP (mmHg)	35.7 ± 9.8	49.5 ± 13.6	3.214	0.003*
PAWP (mmHg)	11.3 ± 4.9	12.9 ± 4.1	1.099	0.278
CO (L/min)	4.6 ± 0.7	5.6 ± 1.4	2.357	0.023*
PVR (Wood) unit	4.8 ± 3.4	7.3 ± 3.6	2.089	0.043*

RAP, right atrial pressure; RVP, right ventricular pressure; sPAP, pulmonary artery systolic pressure; dPAP, pulmonary artery diastolic pressure; mPAP, mean pulmonary artery pressure; PAWP, pulmonary artery wedge pressure; CO, cardiac output; PVR, pulmonary vascular resistance.

* Compared with the control group *P* < 0.05.

**Table 3:** Spearman's correlations between GDF-15 and the other factors in hemodynamic indexes.

Index	*r*	*P*
RAP (mmHg)	0.186	0.222
RVP (mmHg)	0.411	0.005*
sPAP (mmHg)	0.434	0.003*
dPAP (mmHg)	0.545	<0.001*
mPAP (mmHg)	0.522	<0.001*
PAWP (mmHg)	−0.043	0.781
CO (L/min)	0.377	0.011*
PVR (Wood) unit	0.303	0.043*

RAP, right atrial pressure; RVP, right ventricular pressure; sPAP, pulmonary artery systolic pressure; dPAP, pulmonary artery diastolic pressure; mPAP, mean pulmonary artery pressure; PAWP, pulmonary artery wedge pressure; CO, cardiac output; PVR, pulmonary vascular resistance.

* Compared with the control group *P* < 0.05.

### 3.3. Correlation analysis between GDF-15 and indexes of pulmonary arterial morphology

The average levels of Vd, elastic modulus, stiffness index β, lesion length, PAV and NT-proBNP in the GDF-15 <1,200 pg/ml group were lower than those in the GDF-15 ≥1,200 pg/ml group. The average levels of compliance, distensibility, and minimum lumen area were higher than those in the elevated GDF-15 ≥1,200 pg/ml group. The difference between the two groups was statistically significant (*P* < 0.05). There was no statistically significant difference in MWT, WTP, plaque load, plaque volume, and EEM-CSA between the two groups (*P* > 0.05), as is shown in Table 4. Spearman's correlation analysis results showed that the level of GDF-15 was negatively correlated with Compliance, Distensibility and Minimum Lumen Area, and positively correlated with Vd, elastic modulus, stiffness index β, lesion length, PAV and NT-proBNP (*P* < 0.05), as shown in Table 5.

**Table 4:** Results of IVUS measurements of patients in each GDF-15 group $\left(\bar{x}\pm s\right)$.

Index	GDF-15 <1,200 pg/ml Group (*n* = 12)	GDF-15 ≥1,200 pg/ml Group (*n* = 33)	*t*	*P*
Vd (mm)	4.2 ± 0.2	4.4 ± 0.3	2.135	0.039*
MWT (mm)	0.27 ± 0.03	0.29 ± 0.03	1.978	0.054
WTP (%)	12.7 ± 0.8	13.1 ± 0.7	1.632	0.110
Compliance (10–2 mm^2^/mmHg)	24.5 ± 4.6	21.1 ± 5.1	2.027	0.049*
Distensibility (%/mmHg)	2.6 ± 0.4	2.2 ± 0.5	2.428	0.019*
Elastic modulus (mmHg)	83.2 ± 18.7	168.3 ± 16.3	14.900	<0.001*
Stiffness index β	3.9 ± 0.4	4.8 ± 0.6	4.804	<0.001*
Minimum lumen area (mm^2^)	3.1 ± 0.7	2.5 ± 0.6	2.838	0.007*
Lesion length (mm)	19.2 ± 5.7	24.3 ± 4.9	2.957	0.005*
Plaque load (%)	69.8 ± 9.2	74.1 ± 7.9	1.546	0.130
Plaque volume (mm^3^)	165.8 ± 68.5	179.8 ± 75.1	0.565	0.575
EEM-CSA (mm^2^)	13.2 ± 2.9	12.3 ± 3.1	0.875	0.386
PAV (%)	54.1 ± 6.9	59.5 ± 7.2	2.248	0.030*
NT-proBNP, ng/L	1,132.3 ± 447.6	1,697.2 ± 327.0	4.633	<0.001*

Vd, vascular diameter; MWT, mean wall thickness; WTP, percentage of wall thickness; EM-CSA, cross-sectional area of the outer elastic membrane; PAV, percentage of plaque volume; NT-proBNP, N-terminal B-type natriuretic peptide precursor.

* Compared with the control group *P* < 0.05.

**Table 5:** Spearman's correlations between GDF-15 and the other factors in IVUS measurements.

Index	*r*	*P*
Vd (mm)	0.323	0.030*
MWT (mm)	0.086	0.576
WTP (%)	−0.119	0.435
Compliance (10–2 mm^2^/mmHg)	−0.541	<0.001*
Distensibility (%/mmHg)	−0.566	<0.001*
Elastic modulus (mmHg)	0.640	<0.001*
Stiffness index β	0.437	0.003*
Minimum lumen area (mm^2^)	−0.326	0.029*
Lesion length (mm)	0.303	0.043*
Plaque load (%)	0.245	0.104
Plaque volume (mm^3^)	−0.067	0.661
EEM-CSA (mm^2^)	−0.002	0.989
PAV (%)	0.385	0.009*
NT-proBNP, ng/L	0.582	<0.001*

Vd, vascular diameter, MWT, mean wall thickness, WTP, percentage of wall thickness, EM-CSA, cross-sectional area of the outer elastic membrane; PAV, percentage of plaque volume; NT-proBNP, N-terminal B-type natriuretic peptide precursor.

* Compared with the control group *P* < 0.05.

### 3.4. Comparison of prognosis of patients in different GDF-15 groups

As of the end of the study, the average follow-up period of PAH patients was 32.47 ± 1.28 months (mean ± standard deviation). During the follow-up, 8 patients were lost to follow-up due to all-cause mortality, including 2 systemic lupus erythematosus-related PAHs, 1 mixed connective tissue disease-related PAH, 1 systemic sclerosis-related PAH, 2 IPAHs, 1 portal PAH, and 1 congenital heart disease-related PAH.

**Figure 1:**
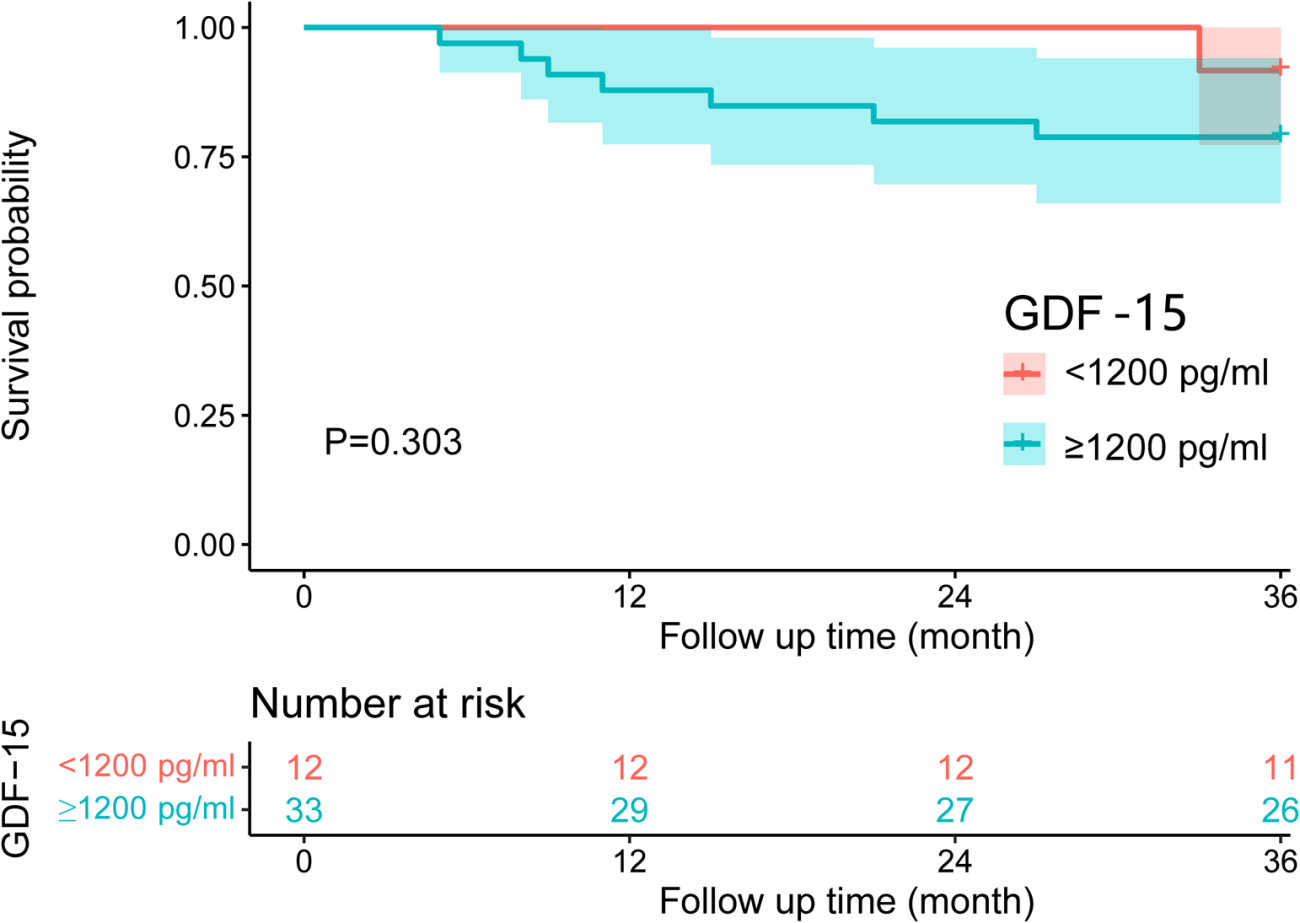
Survival curves of different GDF-15 groups.

The GDF-15 <1,200 pg/ml group had a 1-year survival rate of 100% and a 3-year survival rate of 91.7%. The GDF-15 ≥1,200 pg/ml group had a 1-year survival rate of 87.9% and a 3-year survival rate of 78.8%. Based on the Kaplan Meier method, there was no statistically significant difference in the survival rate between the two groups (*P* > 0.05), as is shown in Table 6 and Figure 1.

**Table 6:** Comparison of survival rates of different GDF-15 groups.

Group	Survival rate		*χ* ^2^	*P* value
	1 year	3 years		
GDF-15 <1,200 pg/ml Group (*n* = 12)	100%	91.7%	1.060	0.303
GDF-15 ≥1,200 pg/ml Group (*n* = 33)	87.9%	78.8%		

## 4. Discussion

Pulmonary arterial hypertension-associated connective tissue disease (PAH-CTD) is a great challenge for both patients and physicians (30, 31), and its common pathological changes are arteriolar intima-media hypertrophy and arteriolar myosification. In order to explore pulmonary vascular abnormalities in patients with PAH, IVUS can often be used to observe the morphological changes of vessels in each layer, measure mechanical properties, and evaluate vascular function through quantitative and qualitative analysis (32). Studies have shown that GDF-15, as an important cardiovascular protective factor, is closely related to the diagnosis and prognosis of many cardiovascular diseases (15). Therefore, in this study, RHC and IVUS were used to detect pulmonary hemodynamics and pulmonary vascular morphology, and ELISA was used to detect serum GDF-15 level to investigate the correlation between serum GDF-15 and pulmonary hemodynamics and pulmonary vascular morphological changes in patients with pulmonary hypertension.

Studies have found that GDF-15 is associated with the severity of pulmonary artery disease and the increase in GDF-15 level is significantly correlated with increased right atrial mean pressure, increased pulmonary capillary pressure, decreased mixed venous oxygen saturation, and increased uric acid and NT-proBNP levels (33). However, the increase in GDF-15 level had no significant correlation with pulmonary artery mean pressure, cardiac index, and cardiac output (34). The results of our study show that there was no statistically significant difference in the average levels of right atrial pressure, pulmonary wedge pressure, and cardiac output between normal GDF-15 group and elevated GDF-15 elevated group. The average levels of right ventricular pressure, pulmonary systolic pressure, pulmonary diastolic pressure, mean pulmonary artery pressure, pulmonary vascular resistance, and other indicators in the elevated GDF-15 group were higher than those in the normal GDF-15 group. This corroborates previous findings and suggests that GDF-15 is associated with the severity of pulmonary artery disease. Pulmonary artery pressure is an index that directly reflects the severity of a patient's condition. The results of this study show that the GDF-15 level is significantly related to pulmonary artery pressure, suggesting the value of GDF-15 in reflecting the severity of the disease. The prognosis of PAH patients depends more on right heart function than pulmonary artery pressure. Right heart index, cardiac output, and NT-proBNP are commonly used indexes to reflect patient heart function. This study shows that GDF-15 level is significantly related to these indices, which indirectly reflects the value of GDF-15 in predicting the right heart function of patients. Pulmonary vascular resistance is a direct cause of PAH and an indicator of the severity of the patient condition. The results of this study show that the GDF-15 level significantly correlates to pulmonary vascular resistance, showing its value in reflecting the severity of the disease. Our research results suggest that the elevated GDF-15 group has higher pulmonary artery pressure and pulmonary vascular resistance, which indirectly reflects that compared with the normal GDF-15 group, the hemodynamic changes in the elevated GDF-15 group are more serious and potentially harmful and need more attention and active treatment. The results of our study differ from other studies, however, a study with a larger sample size is likely to lead to more reliable conclusions.

So far, there is no report on the correlation between GDF-15 and pulmonary vascular morphological changes. We are the first to study the correlation between GDF-15 and morphological changes in pulmonary blood vessels. The average levels of Vd, elastic modulus, stiffness index β, lesion length, PAV, other indexes, the average levels of compliance, distensibility, and minimum lumen area in the elevated GDF-15 group were higher than those in the normal GDF-15 group. But there was no statistically significant difference in MWT, WTP, plaque load, plaque volume, and EEM-CSA between the two groups. This means that pulmonary vascular disease is more serious in patients with elevated GDF-15. Such patients need early and timely prevention and intervention, to improve hemodynamics and even reverse the deterioration of pulmonary vascular performance. Our research confirms that GDF-15 can reflect the severity of morphological changes in pulmonary blood vessels in PAH patients.

In addition, it is reported that GDF-15 is an effective marker of adverse events such as acute coronary syndrome, heart failure, and acute pulmonary embolism (35–37). Recent studies show that GDF-15 is also an independent marker of adverse clinical events in patients with IPAH (16, 33). It can be used together with other markers to assess the risk of diseases and evaluate the therapeutic effect. As per a study, GDF-15 in systemic sclerosis patients with pulmonary arterial hypertension increased significantly and the survival rate decreased compared to those without pulmonary arterial hypertension (17). Laurie et al. conducted a prognostic study on 103 PAH patients (38). Their study confirmed that GDF-15 increased in 76% of patients with pulmonary hypertension, and found that the increased GDF-15 level was related to older age, higher NYHA level, shorter 6 min walking distance, higher average right atrial pressure, and higher NT-proBNP. They also found that GDF-15 was significantly associated with the risk of death or lung transplantation in PAH patients, independent of age and NT-proBNP levels, and had a predictive value. In addition, normal GDF-15 ruled out the risk of death or transplantation within 2 years after diagnosis of pulmonary arterial hypertension. Therefore, GDF-15 may become a promising biomarker, and may be used as a predictor of mortality in PAH patients of various etiologies.

However, due to the lack of tissue specificity, the role of GDF-15 in future biomarker-guided therapy is uncertain. Our results showed that there was no statistically significant difference in survival between the normal GDF-15 group and the elevated GDF-15 group, which may be related to our small sample size, or it may be the elevated levels of GDF-15 are linked to increased inflammation in the more ill patient, or related to a subclass of PAH, which warrants more detailed study in the future. In addition, this study only focused on a single marker, but did not evaluate the combined application value of GDF-15 and existing risk scores. Therefore, in future research, it is necessary to increase the sample size for further study on prognosis correlation, and further research is needed to confirm the predictive ability of GDF-15 and prognosis of patients with pulmonary arterial hypertension.

## 5. Conclusion

Our results show that compared with the normal GDF-15 group, the elevated GDF-15 group had higher pulmonary artery pressure, pulmonary vascular resistance, Vd, elastic modulus, stiffness index β, lesion length, and PAV level, and lower compliance, distension, and minimum lumen area. Furthermore, the elevated GDF-15 group had more severe pulmonary vascular disease lesions, and more serious and potentially harmful changes in hemodynamics and vascular wall morphology. In summary, GDF-15 can reflect the severity and potential harm of hemodynamic changes and morphological changes in patients with PAH, which provides early and timely prevention and intervention for such patients clinically to improve hemodynamics provide evidence.

## Data Availability

The raw data supporting the conclusions of this article will be made available by the authors, without undue reservation.
